# Evaluating a Structured Workflow Guide to Improve Junior Doctor Preparedness for Vascular Surgery Late Shifts: A Prospective Paired Pre-Post Interventional Study

**DOI:** 10.7759/cureus.108905

**Published:** 2026-05-15

**Authors:** Kabir Kohli, Sanjay Patel

**Affiliations:** 1 Vascular Surgery, Guy's and St Thomas' NHS Foundation Trust, London, GBR

**Keywords:** confidence, induction, junior doctors, medical education, perioperative care, preparedness, quality improvement, vascular surgery

## Abstract

Background

Junior doctors covering vascular surgery late shifts, defined locally as shifts covering 12:00 to 22:00, often manage specialty-specific workflows with limited formal induction, including elective admissions, post-operative reviews, medication reconciliation, investigations, and escalation of complications. The objective of this study was to evaluate whether a structured workflow guide improved junior doctors' preparedness and confidence in managing vascular surgery late shifts.

Methods

We conducted a prospective paired pre-post interventional study in a tertiary vascular surgery department. A structured workflow guide was developed and introduced at Foundation Year 1 (FY1) departmental induction. The guide covered shift workflow, electronic admission processes, pre-operative and post-operative review responsibilities, peri-operative medication guidance, investigation requirements, escalation advice, and use of an outpatient accommodation facility. Eight FY1 doctors completed a pre-intervention questionnaire before using the guide and a post-intervention questionnaire after one week. Survey items used a five-point Likert scale across ten preparedness and confidence domains. Supplementary survey items assessed self-reported clerking time and average escalations to senior colleagues per shift per person.

Results

Eight respondents completed both surveys. Median scores improved across all ten domains. The composite preparedness/confidence score increased from 19.5 (interquartile range (IQR) 17.5-21.5] pre-intervention to 46.0 (IQR 43.75-47.5) post-intervention, representing a statistically significant improvement (Wilcoxon signed-rank test, W = 0, p = 0.0078). Supplementary workflow survey items suggested reduced average clerking time from 1.8 to 1.2 hours and reduced average escalations to senior colleagues per shift per person from 5.25 to 3.5. Post-intervention usability scores were high, including median scores of 5.0 (IQR 5.0-5.0) for improved understanding of post-operative review priorities and recommendations to future FY1 doctors.

Conclusions

A structured workflow guide was associated with improved self-reported preparedness and confidence among FY1 doctors covering vascular surgery late shifts. Supplementary survey findings also suggested reduced clerking time and reduced reliance on senior escalation.

## Introduction

Transitioning into specialty-specific on-call and ward-based roles is a challenging part of early United Kingdom (UK) postgraduate medical training after graduation from medical school, including the Foundation Programme. Newly qualified doctors often report gaps in preparedness during the move from medical school into foundation practice, particularly in relation to practical ward systems, local workflows, and confidence in day-to-day clinical responsibilities [1,2]. Induction quality is variable, and poor or inconsistent induction experiences may leave trainees underprepared for the practical demands of a new post [3,4].

These challenges can be amplified when junior doctors begin work in departments with highly specific local processes. Vascular surgery late shifts, defined locally in this study as shifts running from 12:00 to 22:00 hours, may involve a mixture of preoperative elective admissions, postoperative reviews, perioperative medication decisions, specialty-specific investigation requests, and escalation of potentially serious complications. Much of this knowledge is acquired informally from colleagues rather than through structured induction. Targeted induction tools refer to specialty-specific educational resources designed to support junior doctors entering a new clinical environment; examples include handbooks, structured workflow guides, simulation-based preparation, and focused specialty induction resources. Such tools have been shown to improve junior doctor confidence, preparedness, and transition into unfamiliar roles [4-7].

Within our department, incoming FY1 doctors reported uncertainty around several key components of the vascular late shift, including distinguishing between preoperative and postoperative workflows, navigating the electronic admission process, medication reconciliation, perioperative medication withholding, use of outpatient accommodation facilities for suitable elective patients, and recognising complications requiring escalation. Historical feedback from previous FY1 doctors suggested that these issues were persistent across cohorts. In response, we developed a structured workflow guide intended to standardise key tasks and provide a practical reference during shifts.

The aim of this study was to evaluate whether the introduction of this structured workflow guide improved FY1 doctors' preparedness and confidence in managing vascular surgery late shifts.

## Materials and methods

Study design and setting

This was a prospective paired pre-post interventional study conducted within the vascular surgery department at St Thomas' Hospital, Guy's and St Thomas' NHS Foundation Trust, London, UK.

Study population

Participants were FY1 doctors rotating through vascular surgery who were expected to undertake vascular late-shift duties during the study period. The vascular surgery department included six vascular FY1 doctors, with two anaesthetics FY1 doctors also rotating in to cover vascular FY1 late shifts and attending the relevant induction. The wider medical staffing structure included eight senior house officers, 14 registrars, consultant vascular surgeons, clinical nurse specialists, and other multidisciplinary staff. The intervention was targeted at FY1 doctors undertaking late-shift duties and therefore focused on their practical responsibilities and escalation pathways rather than full departmental workforce planning.

Intervention

A structured written workflow guide was developed to support junior doctors covering vascular late shifts (see Appendix A). The intervention was created using existing departmental workflow documents, practical experience from current vascular FY1 doctors, historical feedback from previous FY1 cohorts, and senior clinical input. It was designed as a concise, shift-focused reference resource and was introduced to incoming FY1 doctors during departmental induction for use both during induction and as a practical reference during live clinical shifts.

The guide focused on the practical late-shift responsibilities of FY1 doctors in vascular surgery rather than technical operative details. It covered preoperative elective vascular admissions, postoperative reviews following surgery or interventional radiology, medication reconciliation, perioperative medication withholding, investigation and crossmatch requirements, use of an outpatient accommodation facility for suitable elective patients, electronic admission workflow, escalation pathways, and recognition of common postoperative vascular complications.

The guide clarified the distinction between two common vascular late-shift workflows: preoperative elective admissions and postoperative reviews. Preoperative patients were elective patients attending before a planned procedure, where the FY1 role focused on admission clerking, medication reconciliation, preoperative blood tests and investigations, and ensuring the patient was prepared for theatre or the intended procedure. Postoperative patients were those returning after surgery or interventional radiology who required an overnight bed, where the FY1 role focused on postoperative review, checking the operation or procedure note, reviewing observations and clinical status, ensuring medication reconciliation and post-operative prescribing were correct, checking the documented post-operative plan, and escalating concerns or complications where required.

During vascular surgery late shifts, FY1 doctors escalated concerns to the first-line senior doctor covering vascular surgery, typically a senior house officer, core surgical trainee, or equivalent. Further escalation was to the vascular registrar and, where clinically required, the on-call consultant. The guide included advice on when to escalate routine workflow queries, post-operative concerns, and potential complications.

Data collection

Participants completed a baseline questionnaire before using the guide and a follow-up questionnaire after one week of exposure to the intervention (see Appendix B). Survey items used a five-point Likert scale, where 1 represented strongly disagree and 5 represented strongly agree. The pre- and post-intervention questionnaires assessed preparedness to manage preoperative To Come In (TCI) elective admissions, preparedness to perform postoperative vascular patient reviews, confidence with medication reconciliation, confidence deciding which peri-operative medications should be withheld, confidence ordering appropriate pre-operative investigations and blood tests, confidence using the electronic patient record to admit vascular patients correctly, confidence identifying common vascular postoperative complications requiring escalation, understanding of escalation pathways during a vascular late shift, and overall preparedness to undertake a vascular late shift safely and efficiently.

The post-intervention questionnaire additionally assessed ease of use of the guide during a shift, perceived improvement in understanding of the vascular late-shift workflow, perceived improvement in confidence with peri-operative medication management, perceived improvement in understanding of post-operative review priorities, willingness to recommend the guide to future FY1 doctors, average time taken to clerk a patient including admission tasks and medication reconciliation, and average number of escalations to senior colleagues per shift per person.

For the supplementary workflow outcome, clerking time was defined as the self-reported time required to complete the vascular admission process, including history taking, clinical review where required, documentation, electronic admission tasks, medication reconciliation, and initial investigation requests. The study did not assess documentation quality using the SOAP (Subjective, Objective, Assessment, Plan) framework. Comparable retrospective responses to the supplementary workflow questions were collected from the previous FY1 vascular cohort to provide contextual comparison. Retrospective previous-cohort survey feedback also informed guide development, but was not included in the primary paired pre-post analysis.

Outcomes

The primary outcome was the change in self-reported preparedness and confidence before and after the introduction of the workflow guide. Secondary outcomes were perceived usability of the guide, perceived usefulness of the guide, and willingness to recommend the guide to future trainees. Supplementary workflow outcomes, derived from additional post-intervention survey items, included self-reported average time taken to clerk a patient and average number of escalations to senior colleagues per shift per person.

Statistical analysis

Descriptive statistics were used to summarise survey responses. Because Likert-scale responses are ordinal, individual Likert-scale items are reported as median and interquartile range (IQR). A composite preparedness/confidence score was calculated for each participant by summing responses to questions 1-10, giving a maximum possible score of 50. Because the same eight participants completed both surveys and the outcome data were ordinal, paired non-parametric analysis was performed using the Wilcoxon signed-rank test. Statistical analysis was performed using Microsoft Excel (Microsoft Corporation, Redmond, Washington, United States) with the Real Statistics Resource Pack add-in (https://real-statistics.com/free-download/real-statistics-resource-pack/). A two-sided p-value of <0.05 was considered statistically significant.

The supplementary workflow outcomes were reported descriptively. Given the retrospective nature of the comparator cohort and the small sample size, formal comparative statistical testing was not performed for time-to-clerk and escalation outcomes. Reporting was informed by SQUIRE 2.0 (Standards for QUality Improvement Reporting Excellence), a framework designed to improve the completeness and transparency of reports describing systematic efforts to improve healthcare quality [8].

Governance and registration

This project was undertaken as a departmental quality improvement and educational intervention project within the Vascular Surgery Department at St Thomas’ Hospital. It evaluated a locally developed structured workflow guide designed to support junior doctors during vascular late shifts. Survey participation was voluntary, and responses were anonymised prior to analysis. No patient-identifiable data were collected. The project was registered locally on the clinical audit and quality improvement system (project number: 18887). Formal research ethics committee approval was not required because this was a local quality improvement project involving anonymised staff survey responses.

## Results

Eight respondents completed both pre- and post-intervention surveys. Median scores improved across all 10 preparedness and confidence domains following the introduction of the structured workflow guide. The median pre- and post-intervention preparedness and confidence scores for each survey domain are summarised in Table 1.

**Table 1:** Pre- and post-intervention preparedness and confidence scores IQR: interquartile range

Survey question	Pre-intervention score, median (IQR)	Post-intervention score, median (IQR)	Change in median
I feel prepared to manage preoperative vascular To Come In (TCI) admissions on a late shift.	2.00 (1.00-2.25)	4.00 (3.75-4.25)	+2.00
I feel prepared to perform postoperative vascular patient reviews on a late shift.	2.00 (2.00-2.25)	4.00 (3.75-5.00)	+2.00
I feel confident completing medication reconciliation for vascular admissions.	2.00 (1.75-2.25)	5.00 (5.00-5.00)	+3.00
I feel confident deciding which perioperative medications should be withheld.	3.00 (2.75-3.25)	5.00 (5.00-5.00)	+2.00
I feel confident ordering the correct preoperative investigations and blood tests.	1.00 (1.00-1.25)	4.00 (3.75-4.25)	+3.00
I feel confident using the electronic patient record to admit vascular patients correctly.	1.00 (1.00-1.00)	5.00 (5.00-5.00)	+4.00
I feel confident identifying common vascular postoperative complications that require escalation.	1.00 (1.00-1.25)	4.00 (3.75-5.00)	+3.00
I know when and to whom to escalate concerns during a vascular late shift.	2.50 (2.00-3.00)	5.00 (5.00-5.00)	+2.50
Overall, I feel prepared to undertake a vascular late shift safely.	2.00 (1.00-3.00)	5.00 (5.00-5.00)	+3.00
Overall, I feel prepared to undertake a vascular late shift efficiently.	2.00 (1.75-3.00)	5.00 (5.00-5.00)	+3.00

The composite questions 1-10 score increased from a median of 19.5 (IQR 17.5-21.5) pre-intervention to 46.0 (IQR 43.75-47.5) post-intervention. This represented a statistically significant improvement in the composite score following introduction of the guide (Wilcoxon signed-rank test, W = 0, p = 0.0078).

Usability and acceptability

Post-intervention usability scores were high. Respondents reported that the guide was easy to use during a shift, improved their understanding of the vascular late-shift workflow, and improved confidence in peri-operative medication management. Understanding of post-operative review priorities was rated particularly highly, and respondents strongly supported recommending the guide to future FY1 doctors covering vascular surgery. Post-intervention usability and acceptability outcomes are summarised in Table 2.

**Table 2:** Post-intervention usability and acceptability scores IQR: interquartile range

Post-intervention question	Score, median (IQR)
The guide was easy to use during a shift	4.00 (4.00-4.00)
The guide improved my understanding of the vascular late-shift workflow	4.00 (4.00-4.00)
The guide improved my confidence in perioperative medication management	4.00 (4.00-4.00)
The guide improved my understanding of postoperative review priorities	5.00 (5.00-5.00)
I would recommend this guide to future Foundation Year 1 doctors covering vascular surgery	5.00 (5.00-5.00)

Supplementary workflow outcomes

Supplementary workflow outcomes derived from additional survey questions suggested improvement following the introduction of the structured workflow guide. The average self-reported time taken to clerk a patient, including admission tasks and medication reconciliation, decreased from 1.8 hours in the previous FY1 vascular cohort to 1.2 hours in the post-intervention cohort. The average self-reported number of escalations to senior colleagues per shift per person also decreased from 5.25 in the previous cohort to 3.5 following the introduction of the guide. Supplementary workflow outcomes comparing the previous FY1 cohort and post-intervention cohort are summarised in Table 3.

**Table 3:** Supplementary workflow outcomes

Outcome	Previous Foundation Year 1 cohort	Post-intervention cohort
Average time taken to clerk a patient, hours	1.8	1.2
Average number of escalations to senior colleagues per shift per person	5.25	3.5

## Discussion

This small single-centre pilot prospective study evaluated a structured workflow guide designed to support junior doctors covering vascular surgery late shifts. Following introduction of the guide, self-reported preparedness and confidence improved across all measured domains, with statistically significant improvement in the paired composite questions 1-10 score.

The greatest gains were seen in confidence using the electronic patient record admission process, medication reconciliation, peri-operative prescribing decisions, and overall preparedness to undertake a vascular late shift safely and efficiently. These were areas identified during project development as recurrent sources of uncertainty for incoming trainees. The findings are consistent with previous work suggesting that structured induction resources, handbooks, and on-call preparation interventions can improve confidence, knowledge, and transition into new clinical roles [1,3-7].

Vascular late shifts involve a mixture of preoperative admissions, postoperative reviews, perioperative medication decisions, and local administrative processes that are unlikely to be covered adequately by generic trust induction. The workflow guide evaluated in this study was designed to address that gap by consolidating local practical knowledge into a single, accessible reference. High post-intervention usability scores suggest that the intervention was acceptable to trainees and practical to use in the clinical setting.

The supplementary workflow outcomes add practical context to the findings. The post-intervention cohort reported a reduction in the average time taken to clerk a patient from 1.8 hours to 1.2 hours. This is consistent with the concept that clerking can be affected by delays, interruptions, documentation processes, and system-related tasks [9]. The average number of escalations to senior colleagues per shift per person also fell from 5.25 to 3.5. These descriptive findings should be interpreted cautiously because they were self-reported and used retrospective historical comparator responses, but they suggest that the workflow guide may have reduced perceived workflow burden and reliance on senior input for routine aspects of vascular late-shift work.

This project also has potential relevance beyond vascular surgery. Similar specialty-specific induction resources may be beneficial in other departments where junior doctors are expected to take on unfamiliar, workflow-heavy tasks early in a rotation. Previous studies in emergency medicine, simulation-based on-call preparation, and specialty induction programmes have described similar benefits from structured support tools [5-7].

Limitations

This study has several limitations. It was conducted at a single centre with a small sample size. Outcomes were based primarily on self-reported preparedness and confidence rather than objective clinical process measures. The follow-up period was short, and responses may have been influenced by response bias. In addition, post-intervention responses showed very limited dispersion, which may reflect a ceiling effect or uniformly positive perceptions of the intervention. The supplementary workflow outcomes were also self-reported and, for the historical comparator group, retrospectively recalled, which introduces the possibility of recall bias. However, preparedness, confidence, and perceived workflow burden remain meaningful trainee-facing outcomes when evaluating interventions designed to support transition into new roles.

Future directions

Future work could incorporate larger cohorts, longer follow-up periods, and objective process measures such as completeness of clerking documentation, medication reconciliation accuracy, correct completion of admission tasks, or independently measured time to complete key workflow steps. A multi-centre evaluation would improve generalisability.

## Conclusions

This small single-centre pilot study found that a structured workflow guide was associated with improved self-reported preparedness and confidence among FY1 doctors covering vascular surgery late shifts. Supplementary survey findings suggested reduced clerking time and reduced need for escalation to senior colleagues. These preliminary findings support further evaluation of structured specialty-specific workflow resources, ideally using larger cohorts and objective outcome measures. Although the road to becoming a good vascular surgeon is long and rigorous, we believe a proactive approach and commitment to gradual and continuous self-improvement will make a profound difference in one's growth and success.

## Appendices

Appendix A: vascular late-shift workflow guide content summary

**Table 4:** Vascular workflow guide summary Local contact details, internal cost codes, and operationally sensitive information are not included here.

Parameter	Description
Late-shift overview	Local late-shift hours from 12:00 to 22:00, handover expectations, and core FY1 responsibilities. The guide described 12:00-20:00 as mainly focused on pre-operative admissions and post-operative reviews, and 20:00-22:00 as responding to smartpages, continuing outstanding jobs and reviews, and assisting senior colleagues if required.
Patient workflow distinction	Clarified the different workflows for pre-operative elective admissions and post-operative reviews. Pre-operative patients required clerking, medication reconciliation, investigations, and preparation for theatre/procedure. Post-operative patients required review of documentation, observations, prescribing, medication reconciliation, post-operative plans, and escalation where required.
Pre-operative admissions	Admission clerking, review of pre-operative assessment documentation, blood tests, investigations, crossmatch considerations, medication reconciliation, and preparation for theatre or procedure.
Post-operative reviews	Review of operation/procedure notes, observations, clinical status, post-operative prescribing, medication reconciliation, documented plans, and complications requiring escalation.
Electronic admission workflow	Practical guidance on local electronic patient record admission tasks and ensuring patients were visible on the correct clinical list or workflow.
Peri-operative medication guidance	Advice on identifying documented medication plans, reconciling regular medications, and checking appropriate sources or senior advice when withholding instructions were unclear.
Outpatient accommodation facility	Eligibility and workflow principles for suitable elective patients using an outpatient accommodation facility before admission or procedure.
Escalation and complications	Escalation hierarchy and examples of vascular concerns requiring senior review, including haemodynamic instability, bleeding, limb concerns, uncontrolled pain, sepsis, or significant deterioration.

Appendix B: survey questionnaire

**Table 5:** Survey questionnaire Likert-scale items were scored from 1 to 5, where 1 represented strongly disagree, 2 represented disagree, 3 represented neutral, 4 represented agree, and 5 represented strongly agree. The final two post-intervention items were free-text numerical questions and were not scored on a Likert scale.

Item	Question	Survey timing	Response format
1	I feel prepared to manage preoperative vascular To Come In (TCI) admissions on a late shift.	Pre and post	Five-point Likert scale
2	I feel prepared to perform postoperative vascular patient reviews on a late shift.	Pre and post	Five-point Likert scale
3	I feel confident completing medication reconciliation for vascular admissions.	Pre and post	Five-point Likert scale
4	I feel confident deciding which perioperative medications should be withheld.	Pre and post	Five-point Likert scale
5	I feel confident ordering the correct preoperative investigations and blood tests.	Pre and post	Five-point Likert scale
6	I feel confident using the electronic patient record to admit vascular patients correctly.	Pre and post	Five-point Likert scale
7	I feel confident identifying common vascular post-operative complications that require escalation.	Pre and post	Five-point Likert scale
8	I know when and to whom to escalate concerns during a vascular late shift.	Pre and post	Five-point Likert scale
9	Overall, I feel prepared to undertake a vascular late shift safely.	Pre and post	Five-point Likert scale
10	Overall, I feel prepared to undertake a vascular late shift efficiently.	Pre and post	Five-point Likert scale
11	The guide was easy to use during a shift.	Post only	Five-point Likert scale
12	The guide improved my understanding of the vascular late-shift workflow.	Post only	Five-point Likert scale
13	The guide improved my confidence in perioperative medication management.	Post only	Five-point Likert scale
14	The guide improved my understanding of postoperative review priorities.	Post only	Five-point Likert scale
15	I would recommend this guide to future FY1 doctors covering vascular surgery.	Post only	Five-point Likert scale
16	On average, how long did it take you to clerk a patient, including technical admission tasks and medication reconciliation? Please answer in hours.	Post only	Free-text numerical response
17	On average, how many times per shift did you escalate to a senior colleague for advice or support? Please provide a numerical estimate.	Post only	Free-text numerical response
